# Semisolid State Sintering Behavior of Aluminum–Stainless Steel 316L Composite Materials by Powder Metallurgy

**DOI:** 10.3390/ma12091473

**Published:** 2019-05-07

**Authors:** Kwangjae Park, Dasom Kim, Kyungju Kim, Seungchan Cho, Kenta Takagi, Hansang Kwon

**Affiliations:** 1 Department of Hard Magenets Research, The National Institute of Advanced Industrial Science and Technology (AIST), Shimo-Shidami, Moriyama-ku, Nagoya, Aichi 463-8560, Japan; k.park@aist.go.jp (K.P.); ds-kim@aist.go.jp (D.K.); 2 Department of Materials System Engineering, Pukyong National University, 365, Sinseon-ro, Nam-gu, Busan 48547, Korea; ngm13@ngm.re.kr; 3 Next Generation Materials Co., Ltd., 365, Sinseon-ro, Nam-gu, Busan 48547, Korea; 4 Functional Composites Department, Korea Institute of Materials Science (KIMS), 797, Changwon-daero, Seongsan-gu, Changwon-si 51508, Korea; sccho@kims.re.kr

**Keywords:** aluminum, stainless steel316L, metal matrix composites, intermetallics, spark plasma sintering, microstructure

## Abstract

Aluminum (Al)-stainless steel 316L (SUS316L) composites were successfully fabricated by the spark plasma sintering process (SPS) using pure Al and SUS316L powders as raw materials. The Al-SUS316L composite powder comprising Al with 50 vol.% of SUS316L was prepared by a ball milling process. Subsequently, it was sintered at 630 °C at a pressure of 200 MPa and held for 5 min in a semisolid state. The X-ray diffraction (XRD) patterns show that intermetallic compounds such as Al_13_Fe_4_ and AlFe_3_ were created in the Al-SUS316L composite because the Al and SUS316L particles reacted together during the SPS process. The presence of these intermetallic compounds was also confirmed by using XRD, scanning electron microscopy (SEM), energy dispersive X-ray spectroscopy (EDS), and EDS mapping. The mechanical hardness of the Al-SUS316L composites was analyzed by a Vickers hardness tester. Surprisingly, the Al-SU316L composite exhibited a Vickers hardness of about 620 HV. It can be concluded that the Al-SUS316L composites fabricated by the SPS process are lightweight and high-hardness materials that could be applied in the engineering industry such as in automobiles, aerospace, and shipbuilding.

## 1. Introduction

High-performance materials are required in various sectors of the engineering industry such as in aerospace applications, engineering of robots, and in automobile manufacture [1,2,3]. Moreover, because of depleting oil reserves, increasing efforts are being made to increase the fuel efficiency of engines. One of the methods for increasing the fuel efficiency is decreasing the weight of the materials used in their manufacture. Consequently, in the transfer machine industry, the demand for vehicle body parts made of lightweight materials has been increasing. In recent times, automobile companies have had to increase the fuel efficiency of their manufactured vehicles because of environmental regulations [4]. Moreover, as fossil fuels are getting depleted, the demand for hybrid or electric vehicles has been increasing. To build these high-efficiency and ecofriendly vehicles, high-strength and lightweight materials are needed [5,6]. 

Aluminum alloys are increasingly being used in the aerospace, automobile and shipbuilding industries. These alloys are made by adding various elements (like Cu, Si, Mg, Mn, and Zn) to pure aluminum, which has a low density of 2.7 g cm^−3^, about a quarter of that of iron. In addition, aluminum alloys are used in various industrial fields because of their excellent processability. They are also corrosion resistant due to the formation of a thin surficial aluminum oxide film. However, they have lower mechanical strength than most ferrous materials [7,8,9]. Stainless steel is also used in many industrial fields because of its outstanding corrosion resistance. Stainless steel contains chromium that forms chromium oxide, which protects the surface of these materials. In addition, stainless steel has a higher mechanical strength than aluminum. However, it has a higher density (7.9 g cm^−3^) and it is also more expensive. Moreover, it is quite difficult to process [10,11,12]. 

There are some reports on the characterization and analysis of Al-Fe composites. Sasaki et al. fabricated a bulk nanocrystalline Al-Fe alloy with 5 at.% of Fe (Al–5 at.% Fe), synthesized by mechanical alloying (MA) and spark plasma sintering (SPS). The alloy exhibited a high compressive yield strength of 1 GPa with a plastic strain of 0.3 and consists of coarse α-Al grains that are formed from the powder boundaries and nanocrystalline regions composed of α-Al and Al6Fe phases [13]. Minamino et al. manufactured bulk nanocrystalline Fe–Al–C alloys made by MA, followed by SPS. Three kinds of alloy powders comprising nanocrystalline Fe with 24 at.% Al and X at.% C (where X = 1, 2, 4) were produced by MA using iron and aluminum powder by adding methanol that were subsequently sintered at 1073–1273 K under 64 MPa by SPS. The compacts with 1 and 2 at.% C have superior mechanical properties (e.g., yield strength of 2150 MPa and rupture strain of 0.14 for a compact with 2 at.% C at room temperature) to those of ordinary Fe_3_Al (e.g., yield strength of 380 MPa and rupture strain of 0.12) [14]. Jia et al. investigated Fe_3_Al-based alloys prepared by MA and SPS and the influence of milling time on the properties of these materials. The results show that Fe reacts with Al completely to form Fe_3_Al during the short SPS processing time. The relative densities of the sintered samples were nearly 100%. Bend strength of 1327 MPa and microhardness of 434 [15] were observed. In our previous research [16], we have successfully investigated the Al-X vol.% SUS316L (X = 20, 50, 80) composites fabricated by mechanical ball milling and SPS in the solid state. The Al-50 vol.% SUS316L composite has the highest value of the Vickers hardness (approximately 630 HV). We also proposed that the Al-SUS316L composite could be fabricated in a semisolid state by SPS.

However, most of the reports have investigated the role of low Al or Fe component added in the matrix. To the best of our knowledge, it has not been reported so far how the addition of a high amount of Al or Fe component to the composite materials affect them. In this study, we have fabricated an Al-SUS316L composite with high Al or SU316L content using mechanical ball milling and SPS. Moreover, we have investigated a semisolid state sintering process that allows the creation of a small amount of a liquid phase. In fact, fabrication of semisolid state sintering with high Al or SUS316L content is not commonly reported. Here, we have investigated the Al-SUS316L composites from the perspectives of its microstructure, phase, and mechanical properties. In addition, we try to propose a mechanism of strengthening for the Al-SUS316L composites such as Orowan looping. We have shown that the Al-SUS316L composites combine the advantages both Al and SUS316L.

## 2. Materials and Methods 

Pure Al powder with a mean particle size of approximately 75 μm (AlCO Engineering Co. Ltd., purity 99.7%, particle size below 75 μm, Seosan, Korea) and SUS316L powder with a mean particle size of approximately 60 μm (Dae Kwang Industry Co. Ltd., particle size below 60 μm, Seoul, Korea) were used as the raw materials. The chemical composition of SUS316L was shown in Table 1. Pure Al and 50 vol.% of the SUS316L powder were mixed with heptane in a stainless-steel jar on a ball mill (SMBL-2, SciLabMix™, Programmable Ball Mill, Seoul, Korea) for 12 h at 200 rpm; 75 mL of heptane and alumina balls were used as the process control agents (PCAs). The heptane was evaporated naturally. After ball milling, the composite powder was charged in a WC-Co mold with a mold size of ø 10 mm in vacuum about 1.6 Pa and spark plasma sintered at 630 °C. Subsequently, it was held for 5 min and then pressed at 200 MPa (Fuji Electronic Industrials Co., Ltd., SPS-321Lx, Saitama, Japan) [16].

The morphologies and particle sizes of pure Al, SUS316L, and the Al-50 vol.% SUS316L composite powder were analyzed by a field emission scanning electron microscope (FE-SEM) (TESCAN, MIRA 3 LMH In-Beam, Brno, the Czech Republic) and a particle size analyzer (PSA, Beckman Coulter, LS 13320, Brea, CA, USA), respectively. The density of each sample was measured 10 times by the Archimedes method using a densitometer (ABJ 120-4M, Kern, Balingen, Germany). The X-ray diffraction patterns were measured using an X-ray diffractometer (Rigaku, Ultima IV, Tokyo, Japan) with a Cu Kα radiation source (λ = 1.5148 Å, 40 kV, and 40 mA) in the 2θ range of 20°–80° using a linear detector (Rigaku, D/tex Ultra, Tokyo, Japan). A step size of 0.02° and a scan rate of 0.06° s^−1^ were used. The microstructures of the composites were analyzed by scanning electron microscopy (SEM) (TESCAN, VEGA II LSU, Brno, the Czech Republic) and energy dispersive X-ray spectroscopy (EDS) (HORIBA, EX-400, Tokyo, Japan). The mechanical properties of each sample was measured 10 times according to Japanese Industrial Standard B 7725 and International Organization for Standardization (ISO) 6507-2 using a load of 0.3 kg for 5 s (Vickers hardness tester, Mitutoyo Corporation, HM-101, Kawasaki, Japan) [16]. 

## 3. Results and Discussion

**Figure 1:**
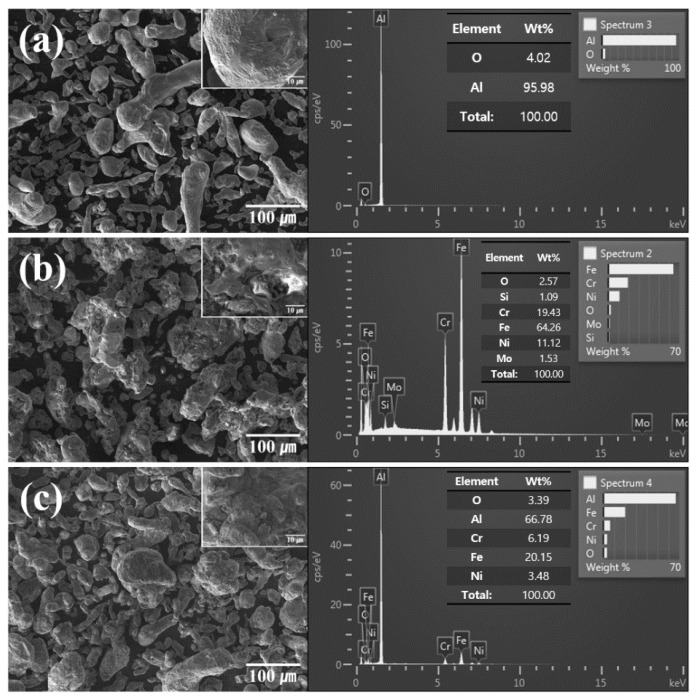
SEM micrograph and EDS spectrum of (**a**) pure Al, (**b**) SUS316L, and (**c**) Al-50 vol.% SUS316L composite powder.

Figure 1 shows the morphologies of pure Al and the SUS316L powders. The pure Al powder has an irregular and rough surface with different particle size distributions (Figure 1a). The SUS316L powder (Figure 1b) also showed an irregular morphology; however, the particles had rougher surfaces than the pure Al particles. The Al-50 vol.% SUS316L composite powder (Figure 1c) also showed a wide size distribution of particles with irregular and rough surfaces, following a trend similar to that of pure Al and the SUS316L powders. However, the surfaces of the composite powder particles were smoother than that of the SUS316L powder because the former was crushed and mixed when subjected to mechanical forces during the ball milling process. The mechanical stress due to the impact of the ball is continuously applied to the composite powders, which causes plastic deformation on the surface of the composite powders during ball milling process. From the view point of morphology, it can be seen that the ball milling process was suitable to manufacture the Al-50 vol.% SUS316L composite powder. The elemental compositions of the pure Al and the SUS316L powders were analyzed by EDS and an oxygen peak was detected. It is well known that a 10–15-nm-thick Al oxide layer typically develops on the surface of alumina because of the reaction between Al and O in air [17,18]. Similarly, in the case of SUS316L, a chromium oxide layer develops on the surface owing to the reaction between Cr and O in air, which prevents rusting [11,19]. Hence, it could be assumed that an Al oxide and a chromium oxide layer developed in the Al, SUS316L composite powder.

**Figure 2:**
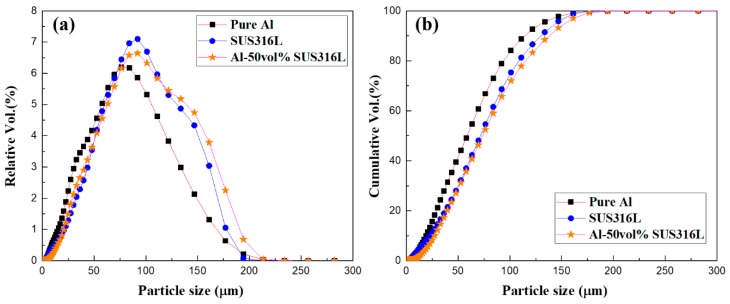
(**a**) Relative and (**b**) cumulative particle size analyses of pure Al, SUS316L, and Al-50 vol.% SUS316L powders.

Figure 2 and Table 2 shows the particle size distribution of pure Al, SUS316L, and Al-50 vol.% SUS316L composite powders. The three powders have relatively similar particle size. In the case of the Al-50 vol.% SUS316L composite powder, the number of particles with size of more than 125 μm was slightly higher. Al is a well-known ductile material and is assumed to first absorb the mechanical energy during the ball milling process and then re-agglomerate with each other [20]. However, the size of the Al-50 vol.% SUS316L composite powder particles was similar to that of pure Al and SUS316L particles, because re-agglomeration rarely occurred in this case. This further corroborates the appropriateness of using ball milling for the fabrication of the Al-50 vol.% SUS316L composite powder, as mentioned above.

**Figure 3:**
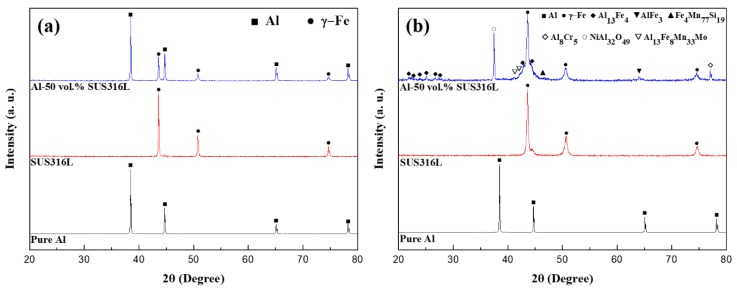
XRD patterns of (**a**) pure Al, SUS316L, and Al-50 vol.% SUS316L powders, (**b**) pure Al and SUS316L bulk and Al-50 vol.% SUS316L composite.

**Figure 4:**
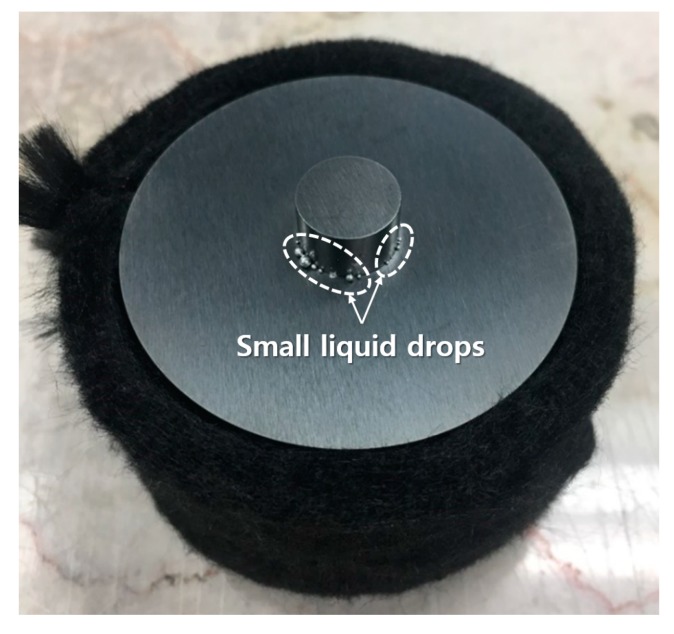
Photograph of cemented mold of semisolid sintered Al-SUS316L composite.

Figure 3 shows the phases of the three powders, their spark plasma sintered bulks, and the composite analyzed using XRD diffraction patterns. The pure Al and SUS316L bulk were manufactured using the same SPS process conditions for comparison with the composite. As shown in Figure 3a, the Al, SUS316L, and Al-50 vol.% SUS316L composite powders show the peaks for Al and γ-Fe only. This means that the Al and SUS316L powders did not react with each other during the ball milling process. However, intermetallic compounds such as Al_13_Fe_4_, AlFe_3_, Fe_4_Mn_77_Si_19_, Al_8_Cr_5_, NiAl_32_O_49_, and Al_13_Fe_8_Mn_33_Mo were detected, as shown in Figure 3b. These intermetallic compounds were assumed to have been produced from the reaction between Al and the components of SUS316L by the micro-plasma during the SPS process. It is also supported by the fact that the peaks of these intermetallic compounds were not present in the diffraction pattern of the Al-50 vol.% SUS316L composite. The micro plasma generated in the SPS process was responsible for the removal of the impurities and oxide layers of the particle interfaces [21,22]. The particles locally react with one another because of the high temperature of the spark plasma [23], and this could lead to the formation of the intermetallic compounds. In our previous research [16], we have successfully fabricated Al-SUS316L composites of various compositions by the SPS process using pure Al and SUS316L powders. These Al-SUS316L composites were spark plasma sintered in the solid state at 600 °C, pressed at 200 MPa, and held for 5 min. All of these Al-SUS316L composites had various kinds of intermetallic compounds. The Al-50 vol.% SUS316L composite has the highest number of intermetallic compounds. In addition, according to the Al-Fe phase diagram, it could be seen that Al and SUS316L can form intermetallic compounds during the SPS process [16]. In this study, we carried out the SPS process at 630 °C, which is lower than the melting temperature of Al in the semisolid state. Figure 4 shows small drops of the liquid phase protruding out of the upper-cemented punch. The liquid phase did not form continuously; instead, as shown in Figure 4, it was generated in small amounts and then stopped. It appears that the temperature inside the mold was over 630 °C during the SPS process due to the presence of the micro-plasma. The SPS device measures the temperature of the cemented mold surface using a thermocouple. During SPS, the temperatures inside and at the surface of the mold could be slightly different [24]. Therefore, it was found that we could not process the materials above 630 °C because of the generation of liquid phases.

**Figure 5:**
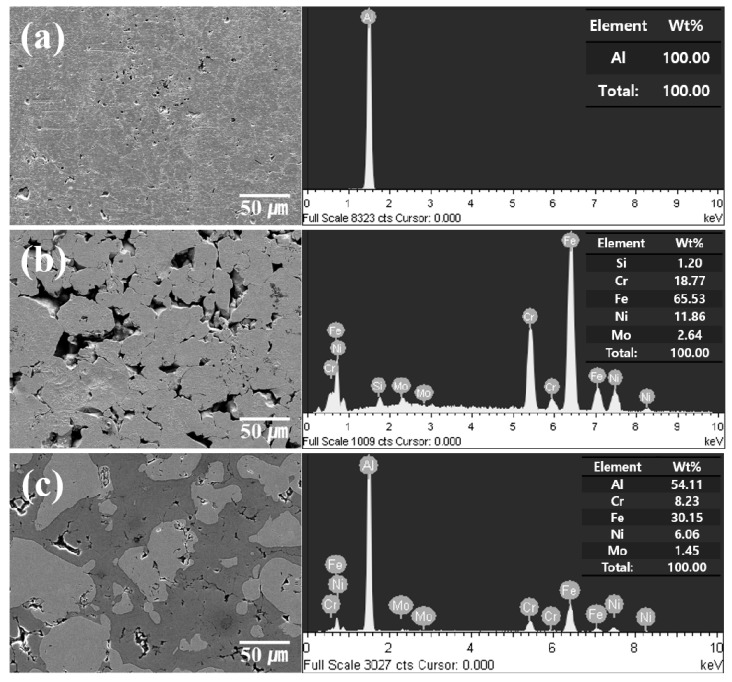
SEM micrographs and EDS analyses of (**a**) pure Al bulk, (**b**) SUS316L bulk, and (**c**) Al-50 vol.% SUS316L composite.

The SEM micrographs and EDS of the pure Al bulk, SUS316L bulk, and Al-50 vol.% SUS316L composite are shown in Figure 5. The pure Al bulk is almost highly dense, as shown in Figure 5a and Table 3. This happened because the sintering temperature was very close to the melting point of Al. However, some grooved-holes looks like pores were detected in Figure 5a. It is assumed that these grooved-holes were created by over-etching performed to observe the microstructure of pure Al bulk. The etchant was mixed with 100 ml of distilled water and 20 g of sodium hydroxide. In other hands, the SUS316L bulk appears to have many pores and much lower density, as shown in Figure 5b and Table 3, because the sintering temperature is much lower than the melting point of SUS316L. Surprisingly, the Al-50 vol.% SUS316L composite has a high density of approximately 97%, as shown in Figure 5c and Table 3. The liquid Al seems to infiltrate into the pores of the Al-50 vol.% SUS316L composite, effectively closing them. In other words, the density of the composite is higher because of the higher density of ductile Al. In addition, once the liquid phase is generated, the densification occurs rapidly owing to the repositioning of the particles in the semisolid state during the SPS process [25]. Thus, liquid phase could infiltrate the pores of the composite, leading to its densification. From the viewpoint of sintering density, semisolid state sintering process was found to be suitable for fabricating the Al-SUS316L composites. The microstructure of the Al-50 vol.% SUS316L composite material comprised two areas: the dark grey and the light grey areas. EDS was used to identify the microstructure of these two specific areas.

**Figure 6:**
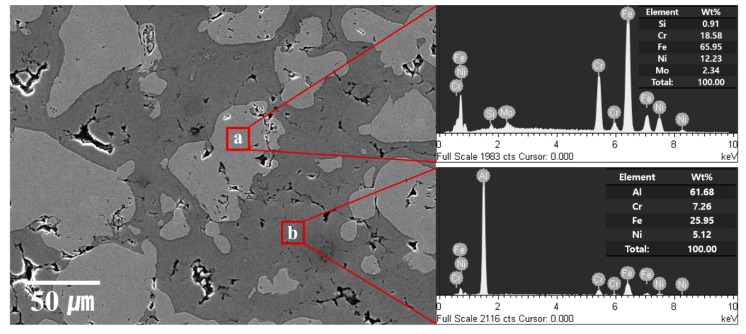
EDS analysis of areas (**a**) and (**b**) of the Al-50 vol.% SUS316L composite.

As shown in Figure 6a, it is clear that the light grey areas mostly consist of the SUS316L phase because the peak of Al was not detected by the EDS. On the contrary, the dark grey areas in Figure 6b are the intermetallic compounds, as we can see the peaks of both Al and SUS316L. These areas were assumed to consist mainly of the intermetallic compounds, which were identified based on the XRD patterns shown in Figure 3. As a whole, structurally, the Al-50 vol.% SUS316L composite consists of the SUS316L phase surrounded by the intermetallic compounds.

**Figure 7:**
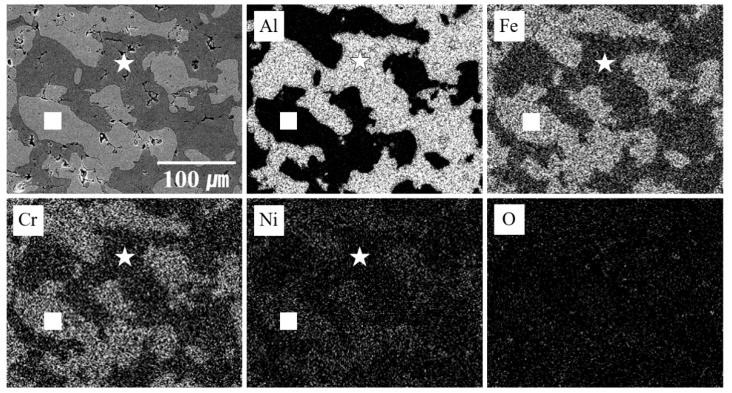
EDS micrographs of Al-50 vol.% SUS316L composite material (inset: □ = SUS316L phase, ☆ = intermetallic compounds).

Figure 7 shows the elemental composition of each phase, as analyzed by EDS mapping to determine the dispersion ratio. The square-marked area is the SUS316L phase because the Al component is nearly zero and the amount of other constituents of SUS316L is high. This square-marked area represents a phase similar to the light grey areas shown in Figure 6a. The intermetallic compounds phase is marked with a star. This star-marked phase has both the Al and SUS316L phases and corresponds to the phase shown by the dark grey areas in Figure 6b. It can be seen that the Al and SUS316L were well dispersed in the Al-50 vol.% SUS316L composite and that the intermetallic compounds surrounded the SUS316L phase, according to EDS mapping. The Vickers hardness was measured to investigate the effect of the intermetallic compounds on the Al-50 vol.% SUS316L composite.

**Figure 8:**
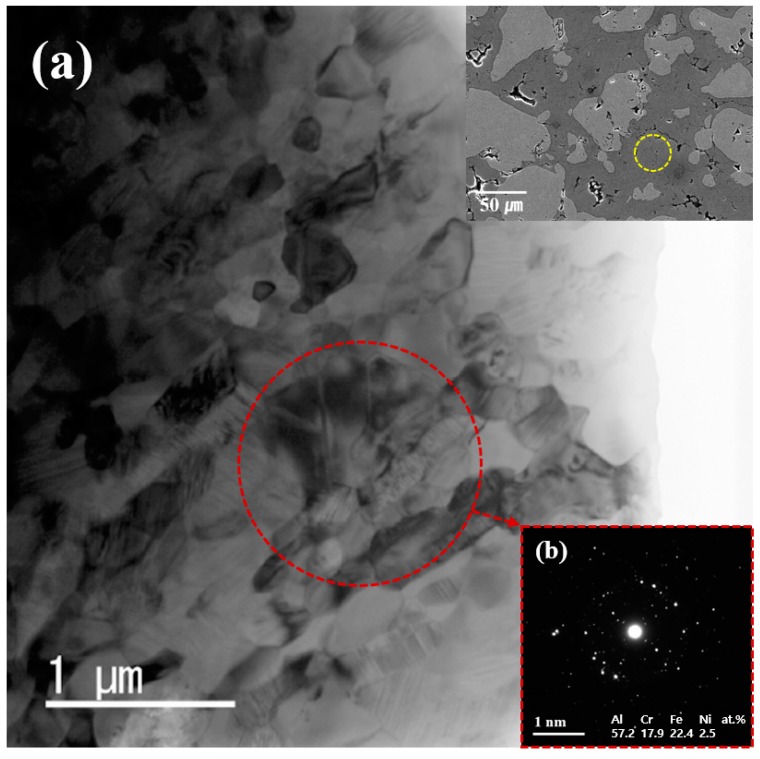
TEM micrographs of the intermetallic compounds region indicated by the yellow circle in inserted Figure 6 and (**b**) Electron diffraction patterns of the region in the red circle in (**a**).

In Figure 8, TEM was used for more precise analysis of intermetallic compounds. Figure 8a shows a TEM micrographs of the intermetallic compounds part in yellow circle in the SEM micrographs of the inserted Al-50 vol.% SUS316L composites. It is assumed that the intermetallic compounds were created as polycrystalline. In addition, it is also confirmed that the aluminum and components of SUS316L were not clustered and uniformly distributed in the intermetallic compounds. Figure 8b shows the electron diffraction pattern of the red circle in Figure 8a. In fact, it is very difficult to analyze a precise quantitative composition of the intermetallic compounds, however, this electron diffraction pattern represents a typical polycrystalline structure and is considered to be an intermetallic compound with a composition similar to Al_2_Fe. As a result, the Al-SUS316L composites were reinforced with intermetallic compounds, and the Al and SUS316L components were homogeneous distributed in a nanosized unit and were created of polycrystalline.

**Figure 9:**
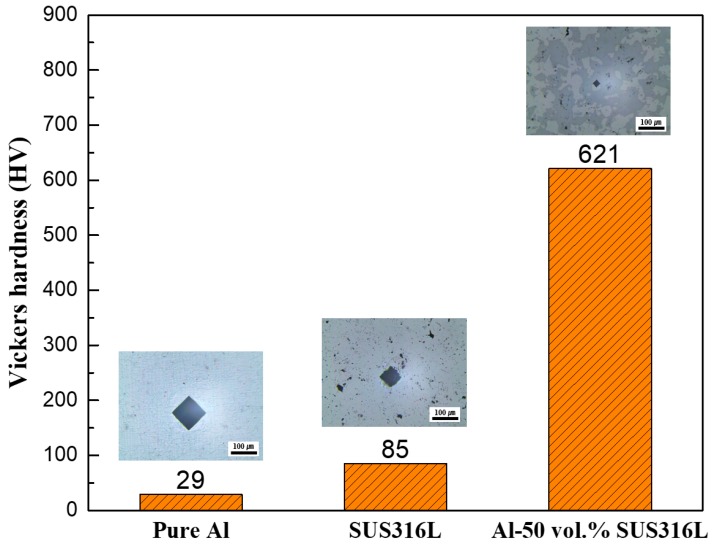
Vickers hardness of the pure Al bulk, SUS316L bulk, and Al-50 vol.% SUS316L composite.

Figure 9 shows the Vickers hardness of the Al-SUS316L composites. The Vickers hardness of the pure Al bulk prepared in this research was similar to that of the generally available pure Al because the sintering temperature was very close to the melting temperature of Al [26]. However, the SUS316L bulk prepared here shows a lower Vickers hardness because of the lower density and porous morphology of the SUS316L bulk due to the lower sintering temperature than melting temperature of steel [27]. Surprisingly, the Vickers hardness of the Al-50 vol.% SUS316L composite was about 620 HV, which is approximately 21 times higher than that of the pure Al and 7.2 times higher than that of the SUS316L. The strengthening mechanism of the Al-SUS316L composite was analyzed by the Orowan looping mechanism [28]. The Al matrix was reinforced with the SUS316L particles and the determined incremental shear strength of the Al-SUS316L composite is shown in Equation (1) [16,29]:

Δτ = Constant × {(μbA^½^)/(r × ln(2r/r_0_)} (1)

where r is the volume equivalent radius of the precipitator (Fe) = 1.593 × 10^−7^ m; r_0_ is the core radius of dislocation = 3.5 × 10^−9^ m; constant = 0.093 for edge dislocation and 0.14 for screw dislocation; b is Burgers vector = 0.286 × 10^−9^ m; is the modulus of rigidity of the matrix (Al) = 8.0 × 10^10^ N m^−2^; and A is the volume fraction of the precipitator (Fe) = 50 [16,28]. The calculated theoretical Δ shear strength (Δτ) was 10.38 MPa for screw dislocation and 6.90 MPa for edge dislocation [16]. However, the strengthening effect in the Al-SUS316L composite cannot be explained by the theoretical Δ shear strength alone because it was considerably low to effect such an increase in the mechanical hardness. The intermetallic compounds seem to be another factor in the strengthening mechanism. One of the intermetallic compounds was Al_13_Fe_4_, which has a Vickers hardness of approximately 691 HV [30]. The hardness, Young’s modulus, and fracture toughness values of homogeneous intermetallic materials such as FeAl_2_ (Fe_6.5_Al_11.5_), Fe_2_Al_5_ (FeAl_2.7_), and FeAl_3_ (Al_13_Fe_4_) are consistent with the proposed phase transformation and phase identification [30]. The presence of these intermetallic compounds was confirmed by the XRD patterns, SEM, and EDS mapping. These intermetallic compounds that have high Vickers hardness were formed between the Al and the SUS316L matrix, which could increase the mechanical hardness of the Al-SUS316L composite. In general, the creation of intermetallic compounds tends to make materials brittle [31,32,33]. However, some research has reported that intermetallic compounds such as Al_4_C_3_ could help to create the strong chemical bonding between Al matrix and carbon nanotubes [34,35]. In these previous researches, it was reported that these strong chemical bonding could effectively transfer the stress in composites [34]. Similarly, the intermetallic compounds assumed to create the strong chemical bonding between Al and SUS316L matrix, which could affect to increase the load transfer. Further, the semisolid state sintering process could also lead to strengthening of the composite. As mentioned above, the densifying behavior observed in the semisolid state sintering process could be responsible for the enhanced mechanical properties of the composite. It is possible that because the Al matrix and the intermetallic compounds were combined in the SUS316L, it increased the bond strength between the matrices. However, only the analysis of other mechanical properties such as elongation and tensile strength and an intensive microstructural analysis by transmission electron microscopy (TEM) are needed to accurately identify the specific strengthening mechanism in the Al-SUS316L composite, which will be our next tasks. 

## 4. Conclusions

We have successfully fabricated Al-SUS316L composites using a mechanical ball milling and semisolid state SPS processes. The Al-50 vol.% SUS316L composite has a Vickers hardness of approximately 620 HV. Intermetallic compounds such as Al_13_Fe_4_ and AlFe_3_ were formed by the reaction between Al and SUS316L. The presence of these intermetallic compounds was confirmed by XRD patterns, SEM (EDS), and EDS mapping results of the Al-SUS316L composite. It is possible that the formation of these intermetallic compounds having high mechanical hardness is an important factor in the strengthening mechanism of the Al-SUS316L composite. Moreover, the semisolid state sintering process could also be a contributing factor to the strengthening effect of the composite. More intensive studies such as TEM, tensile strength analysis, and corrosion resistance test are needed to clearly identify the factors influencing the mechanical strength of the composite. Thus, the Al-SUS316L composite fabricated by the SPS process could be applied as a lightweight and high-hardness multifunctional composite material and used in the engineering industry.

## Figures and Tables

**Table 1:** Chemical composition of SUS316L.

Sample	Chemical Composition (mass %)							
	C	Ni	Cr	Mo	Si	Mn	P	S
SUS316L	0.02	13	17	2.5	1.0	2.0	0.045	0.03

**Table 2:** Particle size distribution of pure Al, SUS316L and Al-50 vol.% SUS316L composite powders.

Sample	Particle Size (μm)		
	D10	D50	D90
Pure Al	20.8	64.3	125.4
SUS316L	25.0	78.6	142.8
Al-50 vol.% SUS316L	29.9	80.8	151.4

**Table 3:** The physical properties of Al, Ti, and Al-Ti composites.

Sample	Density			Particle Size (μm)	Vickers Hardness (HV)
	Theoretical Density (g cm^−3^)	Experimental Density (g cm^−3^)	Relative Density (%)		
Pure Al	2.70	2.65 ± 0.1	98.4 ± 0.2	76.4 ± 10	29 ± 3
SUS316L	7.98	6.45 ± 0.1	80.8 ± 0.1	92.1 ± 11	84 ± 9
Al-50 vol.% SUS316L	5.34	5.19 ± 0.1	97.2 ± 0.2	93.3 ± 13	621 ± 121
